# SNF TO HOME: OUTCOMES OF THE SNF RE-ENGINEERED (RED) DISCHARGE PROCESS

**DOI:** 10.1093/geroni/igz038.3035

**Published:** 2019-11-08

**Authors:** Lori L Popejoy, Yan Wang, David Mehr, Amy A Vogelsmeier, Bonnie Wakefield, Colleen M Galambos, Greg Petroski

**Affiliations:** 1 University of Missouri, Columbia, Missouri, United States; 2 University of Wisconsin Milwaukee, Milwaukee, Wisconsin, United States

## Abstract

Re-engineered (RED) Discharge Process has been effective in hospitals but little tested in SNFs. This study tested the effect of an 18-month adapted RED implementation in four Midwestern SNFs on 30, 60, 180 day hospital, SNF, and emergency department (ED) readmissions. The sample included 1026 SNF residents discharged to the community during 2013 (n = 526) and 2015 (n = 500). We used Medicare claims Data and Minimum Data Set (3.0) File to identify SNF admissions and readmissions to SNF, acute care, or ED. Data were linked across all four facilities by year and compared using appropriate statistics for descriptive, comparison, and regression tests. Residents discharged in 2013 had statistically significantly higher Charlson Comorbidity Scores 4.65 vs 3.97 in 2015 (p < 0.01), while those discharged in 2015 had significantly worse cognitive status, mood, and ADL self-performance. Overall, rates and number of 30, 60-day, 180 rehospitalization, SNF readmission, ED visits with rehospitalization were lower for all 2015 compared to 2013, but not statistically significantly different, except for the number of 180-day rehospitalizations in 2015. Likewise, the only statistically significantly different result was in the regression model was 180 day rehospitalizations. However, in the two facilities that had the best uptake of the intervention, numbers of readmissions were lower at 60 and 180 days (p=.0106 and .0013, respectively for regression results). SNF RED may be a useful tool for SNFs to use to reduce rehospitalizations after discharge; however, benefit depends on the degree of adoption of the intervention.

